# Supplementary material for: Data Independent Acquisition to Inform the Development of Targeted Proteomics Assays Using a Triple Quadrupole Mass Spectrometer
Source: bioRxiv. 2024 May 30:2024.05.29.596554. Preprint. [Version 1] doi: 10.1101/2024.05.29.596554 (PMC11160738; doi:10.1101/2024.05.29.596554)
Supplement: Supplement 1 [file media-1.pdf]

SUPPLEMENTAL FIGURES.

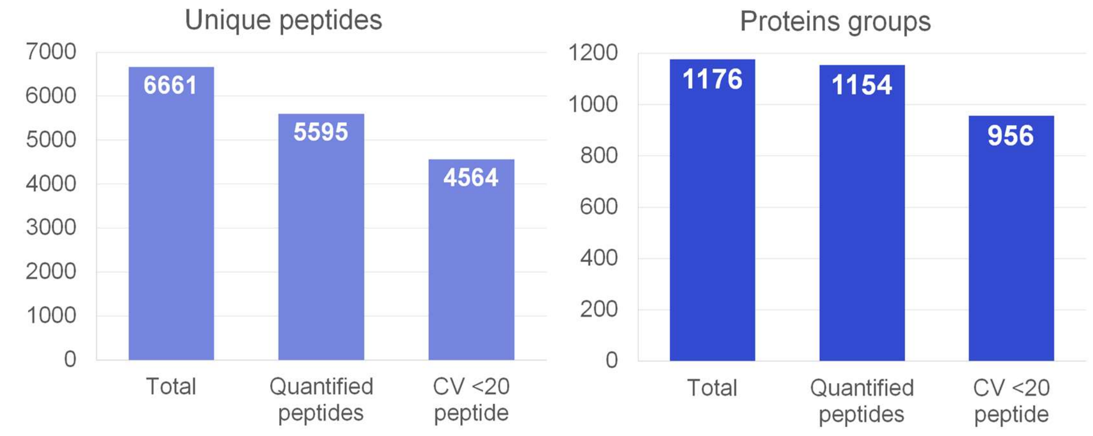

Supplemental Figure 1. Number of protein and peptide detections by DIA experiments. The total number of detections from 3 replicate experiments is plotted for both proteins and peptides (Total). The number of proteins with at least one peptide with at least 3 interference-free product ion transitions (Quantified Peptides). Peptides were filtered for those that exhibit a peak area CV of less than 20% over the 3 GPF libraries (CV <20 peptide).

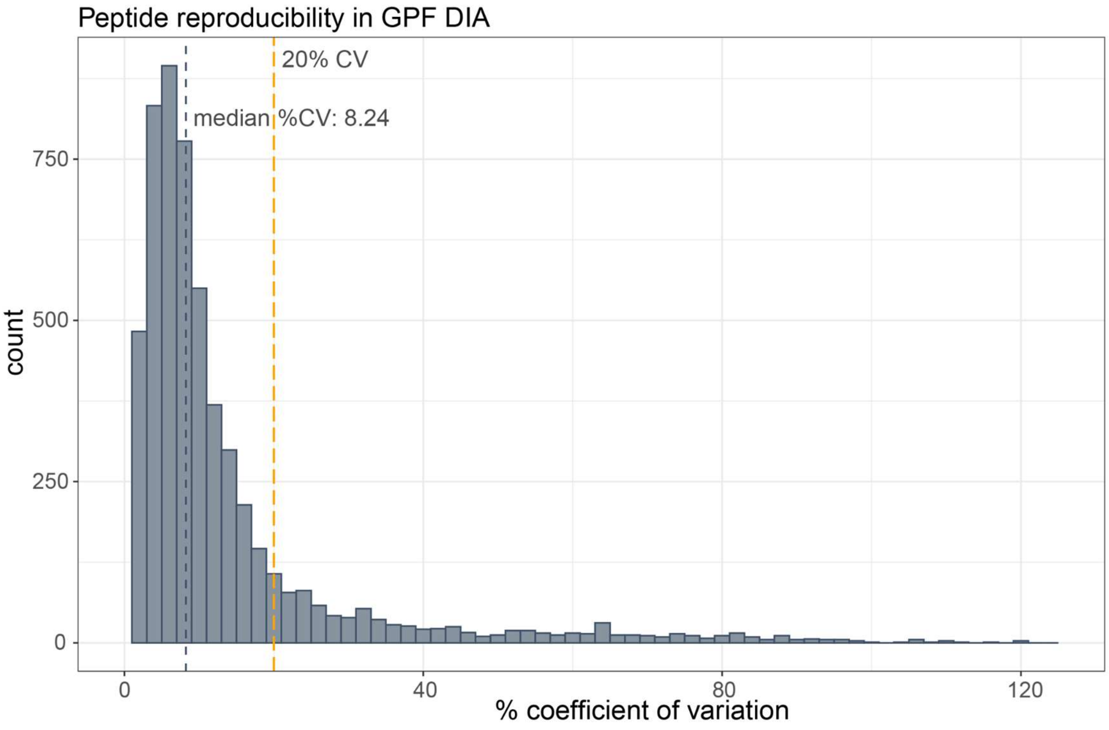

Supplemental Figure 2. Peptide reproducibility across 3 gas-phase fractionated DIA experiments. The percent coefficient of variation for all peptides detected by DIA with at least 3 interference-free transitions. The median CV is 8.24%, below the typical threshold of 20% as indicated by the dashed line.

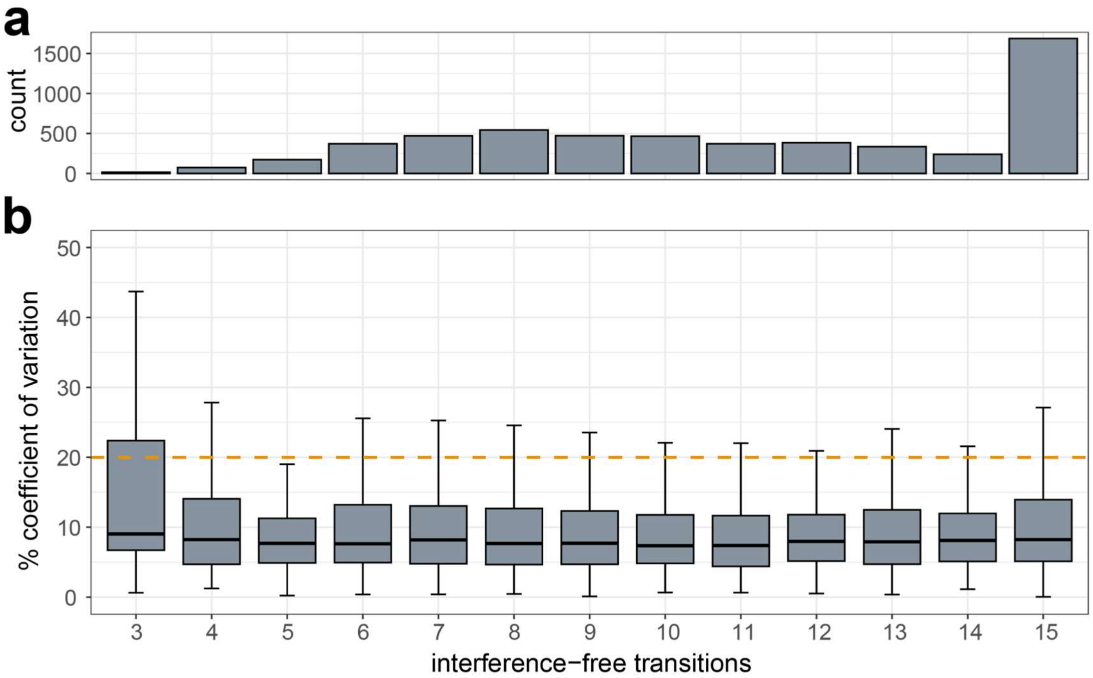

Supplemental Figure 3. Reproducibility is improved with the number of product ion transitions detected per peptide. Using EncyclopeDIA we determine which transitions are interference-free. For peptides that have at least 3 interference-free transitions we extract data in Skyline for quantitation. a) The number of interference-free product ions for each peptide detected by GPF-DIA. The majority (%) of peptides quantified contain more than 5 product transitions. b) The percent coefficient of variation (%CV) from 3 replicate gas-phase fractionated narrow window DIA experiments. The median %CV decreases with an increased number of interference-free transitions per peptide.

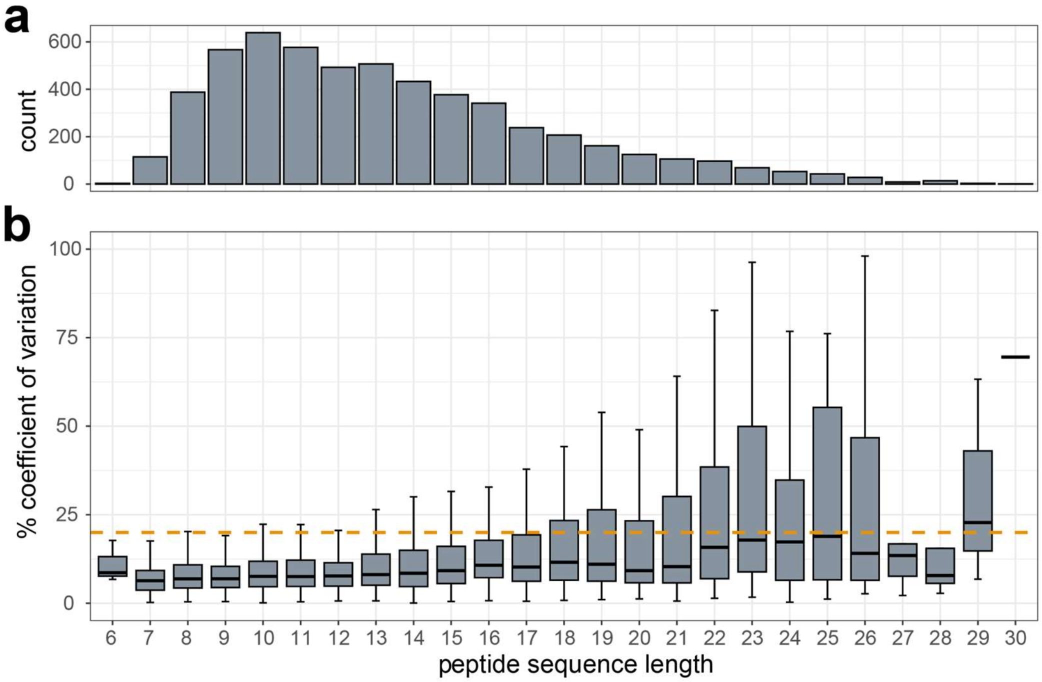

Supplemental Figure 4. Reproducibility of peptides decreases with increasing sequence length. a) The distribution of peptides found in the GPF-DIA by their peptide sequence length. b) The percent coefficient of variation (%CV) from 3 replicate gas-phase fractionated narrow window DIA experiments. The median %CV increases as sequence length increases.
